# Bauxite mine and alumina refinery workers: mortality and cancer risk

**DOI:** 10.1093/occmed/kqae069

**Published:** 2024-09-11

**Authors:** N Kinsman, A Del Monaco, C Dimitriadis, S Xie, G Benke, M R Sim, K Walker-Bone

**Affiliations:** School of Public Health & Preventive Medicine, Monash University, Melbourne, Victoria, Australia; School of Public Health & Preventive Medicine, Monash University, Melbourne, Victoria, Australia; School of Public Health & Preventive Medicine, Monash University, Melbourne, Victoria, Australia; School of Public Health & Preventive Medicine, Monash University, Melbourne, Victoria, Australia; School of Public Health & Preventive Medicine, Monash University, Melbourne, Victoria, Australia; School of Public Health & Preventive Medicine, Monash University, Melbourne, Victoria, Australia; School of Public Health & Preventive Medicine, Monash University, Melbourne, Victoria, Australia

## Abstract

**Background:**

Aluminium industry workers are at risk of long-term health consequences.

**Aims:**

To investigate mortality and cancer incidence in bauxite mine and alumina refinery workers.

**Methods:**

A pre-existing cohort of workers was re-linked with the Australian National Death Index, and the Australian Cancer Database to provide additional death (7 years) and cancer (9 years) data. Standardized mortality ratios (SMRs) and standardized incidence rates (SIRs) were estimated by job category, duration of employment and time since first employment.

**Results:**

Linkage was performed for 6935 (6207 male) workers. Compared with the general population, there was a reduced or similar risk of death for mine/refinery workers for all causes except mesothelioma which was increased amongst male production workers [SMR 2.42, 95% CI 1.11–4.60]. Mesothelioma incidence was also increased amongst males [SIR 2.50, 95% CI 1.60–3.71]. Male office workers had a greater incidence of prostate cancer [SIR 1.30, 95% CI 1.06–1.57] and thyroid cancer [SIR 3.47, 95% CI 1.66–6.38]. Melanoma incidence was increased in female office workers [SIR 2.27, 95% CI 1.36–3.54]. Lip cancer incidence was increased in male maintenance/production workers [SIR 2.04, 95% CI 1.02–3.65]. Overall cancer incidence was otherwise similar to the general Australian population.

**Conclusions:**

Overall risk of death and incidence of cancer for bauxite mine and alumina refinery workers was similar to the general population. Incidence and risk of death from mesothelioma were higher, likely due to historic asbestos exposure in this and other industries. The increased risk of melanoma, lip, prostate and thyroid cancers requires further investigation.

## Teaser

This cohort study of 6935 Australian bauxite mine and alumina refinery workers presents mortality and cancer risk data. A substantial healthy worker effect was demonstrated. Excess mortality rates were found for mesothelioma amongst male employees, particularly production workers. Excess incident cases of mesothelioma were seen amongst male and female workers; melanoma amongst female workers; and lip, prostate and thyroid cancer amongst male workers. Sun protection measures are essential. Further thyroid cancer investigation is required.

## Introduction

Aluminium is a highly versatile metal and an essential component of many commercial and domestic products. Its production has three stages: bauxite mining, refining bauxite into alumina and smelting the alumina into aluminium [1]. Australia has large natural deposits of high-grade bauxite (31% of global production) [2]. Approximately 70% of Australian mined bauxite is refined into alumina in Australia and then exported [2], whilst 14% of the alumina is smelted in Australia (3% of global production) [2]. There are approximately 14 500 workers currently employed in the Australian aluminium industry, inclusive of mining, refining and smelting [3].

Bauxite mining and alumina refining are associated with exposure to a range of hazards [4] including bauxite dust (containing silica), caustic mist and alumina dust, which may increase the risks of respiratory diseases. It is thought that bauxite dust is biologically inert with previous research suggesting it does not cause adverse health effects in workers [4–8]. Diesel fumes are another potential hazard in bauxite mining (also implicated as carcinogenic) although bauxite mine workers have limited exposure due to surface rather than underground mining, ventilation and enclosed vehicle cabins [6]. Maintenance workers are potentially exposed to additional hazards including welding fumes, asbestos (in pipe lagging) and synthetic mineral fibres [1]. Overall, the International Agency for Research on cancer (IARC) has classified working in the aluminium industry as a class I carcinogen [9], although this refers to exposures in the smelters and not the mines and refineries.

The Healthwise Study of smelter, refinery and mining workers in the aluminium industry commenced in 1995 to evaluate long-term health risks. A previous linkage of mine/refinery workers in 2002 found an increased mortality risk from mesothelioma and an increased incidence of mesothelioma and melanoma [10]. However, given the long latency of many cancers, another linkage was performed in 2016 to further evaluate the risks. The results of the re-linkage for Healthwise aluminium smelter workers found an increased risk of death from mesothelioma, lung, prostate and liver cancer, and an increased incidence of overall respiratory cancers [11]. The aim of this analysis was to evaluate risks in mining and refinery workers participating in the Healthwise Study.

## Methods

This paper will report on an analysis of a sub-population of the Healthwise study, a longitudinal cohort of primary aluminium industry workers from three bauxite mines, three alumina refineries and one shipping terminal in Western Australia (WA), the characteristics of which have been described previously [10].

Briefly, the cohort was assembled from three sources: a cross-sectional respiratory health survey performed 1995/96 (*n* = 3328, 48%); an inception cohort study of respiratory health (employment commenced between 1995 and February 2000 [531, 7.7%]); and ex-workers who worked on or after 1 January 1983 and left before the 1995/96 survey (*n* = 3076, 44.3%). Eligibility was based on having worked for 90 days or more, with at least 1 day on or after 1 January 1983.

Participants in the 1995/96 cross-sectional survey and inception cohort study were interviewed and demographic, smoking and job history information was collected at the time of recruitment (job histories were updated, prior to the 2016 linkage, from personnel files in company records). For workers who left employment before the 1995/96 survey, smoking records and a full job history (start/end date, site, operating centre, department and job title) were extracted from personnel files. Workers were classified based on job histories as ever office (mainly unexposed); ever maintenance (irregularly exposed) or ever production (regularly exposed) with the number of years employed in each role calculated [10,12]. Participants missing a job history were excluded from analyses by work classification.

The Australian Institute for Health and Welfare (AIHW) has administered Australian national registries of deaths and cancers from 1983 onwards: the National Death Index (NDI) [13] and Australian Cancer Database (ACD) [14]. Data linkage was undertaken with the most recently available data at the time of analysis (30 November 2016 for deaths and 31 December 2014 for cancer registration). As work sites were all located in WA, linkage was also undertaken to the WA Cancer Registry (WACR) [15]. The WACR provided complete cancer data until the end of 2016 enabling maximization of cancer ascertainment [16], and allowing additional years of follow-up for cancer incidence.

The AIHW used probabilistic linkage to the NDI and ACD to identify possible cancer and death matches, using personal identifiers (surname, first name, birth date), death date (if known) and last contact date (last date of employment). Multiple passes were made of the data using variations of the identifiers and possible matches were supplied with a probability of being ‘true’. Proposed death data matches were independently reviewed by two researchers with disagreements resolved by a third. For privacy reasons, cancer matches were reviewed by registry data custodians, and only ‘highly certain’ matches were released. The underlying cause of death was coded to the International Classification of Diseases, Ninth Revision (ICD-9) [17] until 1996, or Tenth Revision (ICD-10) [18] after 1997, and all cancer incidence records were coded to ICD-10.

Person-years at risk commenced 91 days after the date of first employment, or 1 January 1983, whichever was later. For the duration of employment, follow-up commenced from the date of first employment in any work category (offset by 90 days), or 1 January 1983, whichever was later. Duration of employment in maintenance or production was calculated: never; >3 months and <10 years; >10 years and <20 years; and >20 years. Time since first employment was calculated as >3 months to <20 years; >20 years to <40 years; and >40 years. Person-years were calculated until the end of follow-up: 30 November 2016 for death or 31 December 2016 for cancer, or until the date of death, whichever was earliest. Only primary incident malignant cancers were included in analyses, but cohort members remained at risk of primary cancer at different anatomical sites and each individual cancer was counted.

The expected numbers of cancers and deaths based on 5-year age groups and sex-specific rates were calculated from population data. For the additional years of cancer follow-up obtained from the WACR [15], the 5-year average of the 2009–2014 national population rates was used. Standardized mortality ratios (SMRs) and standardized incidence ratios (SIRs) with 95% confidence intervals (CIs) were calculated for overall death and cancer, and for major cancer and death categories. Analyses were carried out using Stata version 15.1 (StataCorp. 2017. College Station, TX).

This study was approved by the Monash University Human Research Ethics Committee (Project ID: 9941) and access to national cancer and death data was approved by the AIHW Ethics Committee. The Department of Health WA Human Research Ethics Committee granted approval to access WA cancer data.

## Results

In total, 6935 workers (6207 males, 728 females) were included in the study. Of these, 43 (0.7%) workers were excluded from the work category analysis because job history was missing. At linkage in 2016, the mean age was 61 years (males) and 58 years (females). Characteristics of cohort members are summarized in Table 1.

**Table 1.** Characteristics of the 6935 eligible mine/refinery workers

Characteristics	Males *n* (%)	Females *n* (%)	Total
Number of employees	6207 (90)	728 (10)	6935
Age started at mine/refinery, mean (SD) years	29.11 (9)	28.91 (8)	–
Age at end of follow-up, mean (SD) years	60.96 (12)	57.56 (11)	–
Currently employed end 2016	1353 (22)	90 (12)	1443 (21)
Duration of employment			
<5 years	1126 (18)	272 (37)	1398 (20)
5–9 years	730 (12)	151 (21)	881 (13)
10–19 years	1476 (24)	161 (22)	1637 (24)
≥20 years	2875 (46)	144 (20)	3019 (44)
Year started at mine/refinery			
Before 1970	389 (6)	4 (1)	493 (7)
1970–79	1739 (28)	102 (14)	1841 (27)
1980–89	2854 (46)	347 (48)	3201 (46)
1990–99	1225 (20)	275 (38)	1500 (22)
Smoking			
Never smoker	1997 (32)	272 (37)	2269 (33)
Current smoker	1559 (25)	136 (19)	1695 (24)
Former smoker	1554 (25)	91 (12)	1645 (24)
Non-smoker^a^	298 (5)	38 (5)	336 (5)
Unknown	799 (13)	191 (26)	990 (14)
Person-years (follow-up until December 2016)	172 617	19 511	192 128
Average follow-up (years)	27.8	26.8	27.7
Mortality (follow-up until November 2016)			
Deaths	842 (14)	38 (5)	880 (13)
Cancer incidence (follow-up until December 2016)			
Cancers	1150 (18)	95 (13)	1245 (18)

^a^Non-smoker from employment records (cannot rule out previously smoked).

The analysis of mortality for major and specific causes of death amongst male mine and refinery workers by work category is summarized in Table 1a (available as Supplementary data at *Occupational Medicine* Online) and, for female workers, in Table 1b (available as Supplementary data at *Occupational Medicine* Online). Compared with the Australian general population, there was a reduced risk of all-cause death (SMR 0.75, 95% CI 0.70–0.81) and death from most major causes (nervous system disorders had a similar risk). There were also reduced mortality rates for most specific causes, including circulatory and respiratory diseases, and accidental death. Male mine/refinery workers had an increased risk of death from mesothelioma, most notably amongst those who had ever worked in production (SMR 2.42, 95% CI 1.1–4.60). Analyses for female categories with sufficient numbers showed a reduced risk of death amongst female workers with all-cause mortality rate (SMR 0.72, 95% CI 0.51–0.98).

Table 2 (available as Supplementary data at *Occupational Medicine* Online) summarizes the mortality rates for the major and specific causes of death amongst males by duration of employment in maintenance or production. Elevated risks of death from mesothelioma were found across all durations of employment but were significant amongst those who worked for >3 months and <10 years (SMR 4.75, 95% CI 1.91–9.78). For all other causes of death, and any duration of employment, workers had a similar or significantly lower risk of death, compared with the general Australian population. Additional analyses were performed using time since first employment instead of employment duration (data not shown). Similar findings were obtained, with an elevated risk of mesothelioma seen in workers first employed between 20 and 40 years ago (SMR 2.83, 95% CI 1.46–4.94) but not for workers first employed over 40 years ago (SMR 0.84, 95% CI 0.02–4.70) (the latter based on <3 observed cases). Similar, or reduced, risk of death was seen for all other causes across all time since the first employment categories.

Comparison of cancer incidence rates amongst male and female mine/refinery workers with the general Australian population showed many similarities (Table 3a and 3b, available as Supplementary data at *Occupational Medicine* Online). However, the incidence of melanoma was higher, as was the incidence of mesothelioma (SIR 2.50, 95% CI 1.60–3.71) and (SIR 12.73, 95%CI 1.54–45.98) amongst males and females, respectively (although there were <3 cases in females). In addition, male workers had a higher incidence of prostate cancer (SIR 1.14, 95% CI 1.03–1.27), particularly amongst ever office workers (SIR 1.30, 95% CI 1.06–1.57), and there was an increased risk of thyroid cancer (SIR 2.05, 95% CI 1.25–3.17), also driven predominantly by increased rates amongst ever office workers (SIR 3.38, 95% CI 1.54–6.41). Male workers had a lower incidence of respiratory and intrathoracic cancers, and lung cancer (SIR 0.80, 95% CI 0.65–0.98).

Additional analysis of cancers with increased SIR was repeated using WA population rates (data not shown). Thyroid cancer SIRs remained significant; however, increased melanoma rates amongst male workers were attenuated and became nonsignificant (SIR 1.16, 95% CI 0.99–1.35) but those amongst females remained elevated (SIR 1.97, 95% CI 1.20–3.04).

Exploring cancer incidence by employment duration (Table 4, available as Supplementary data at *Occupational Medicine* Online), males who had never worked in maintenance or production were at greater risk of prostate cancer (SIR 1.37, 95% CI 1.07–1.72), and thyroid cancer (SIR 5.15, 95% CI 2.22–10.14). Workers employed in production or maintenance for >3 months and <10 years had a higher incidence of lip cancer (SIR 2.04, 95% CI 1.02–3.65) but no increased risk was seen amongst those employed for longer periods. Elevated risks of mesothelioma were seen in those employed >3 months but <10 years (SIR 5.68, 95% CI 2.83–10.16). Likewise, elevated risks of melanoma were seen in those employed >3 months and <10 years (SIR 1.36, 95% CI 1.01–1.78). When these analyses were repeated by time since employment (data not shown), results were very similar: elevated risks of melanoma were found for time since first employment >3 months and <20 years (SIR 1.53, 95% CI 1.14–2.00) and for mesothelioma amongst those with time since employment >3 months and<20 years (SIR 4.80, 95% CI 1.76–10.45), and 20–40 years (SIR 2.64, 95% CI 1.45–4.44).

## Discussion

Amongst male workers employed in the bauxite mines and alumina refineries, we found an overall lower risk of death from all causes, all malignancies and a range of specific causes including accidents, circulatory diseases, respiratory diseases, digestive and urinary diseases. Amongst female workers, despite small numbers, there was a reduced risk of death from all causes. Importantly, workers exposed to bauxite and silica dust, caustic mist and alumina did not show excess risk of death from respiratory diseases or lung cancer. However, an excess risk of death from mesothelioma was found amongst male workers, with a more than doubling of the risk amongst those who ever worked in production. The reduced risks of death seen in the cohort are most likely explained by the healthy worker effect. Also consistent with this effect, rates of incident cancers amongst workers were similar, or reduced, compared to those found in the general Australian population. However, an excess risk of incident mesothelioma and melanoma was found amongst male and female workers and an excess risk of prostate, lip and thyroid cancers was found amongst male workers.

Excess incident cases of mesothelioma were seen amongst male and female workers in the current study, particularly amongst maintenance/production workers; the highest risks of mesothelioma were seen amongst those with the shortest duration of employment (Table 2 and 4, available as Supplementary data at *Occupational Medicine* Online). The main cause of mesothelioma is asbestos exposure. Bauxite was mined from areas that do not contain asbestos in the ore. In the refineries, asbestos removal began in the early 1980s, with some asbestos-containing material remaining in situ under an asbestos management plan. Additional data were extracted from the Western Australia Mesothelioma Registry [19], which collects histories to determine the most likely exposure events. Five cases could be attributed to likely asbestos exposure whilst employed at the refineries and seven cases had exposure at Wittenoom, a town where substantial asbestos mining occurred in the past. The association with short duration of employment could be explained by workers with previous work exposures, recently joining the refinery. Compared to the results of the previous linkage [10], the SIR has decreased (2.80 [CI 95% 1.75–4.81] to 2.50 [CI 95% 1.60–3.71]), suggesting exposure peak (and subsequent cases) has been reached. Amongst male employees, an excess risk of death from mesothelioma was also found, particularly amongst ever production workers of less than 10 years employment duration.

Melanoma is a skin cancer associated with ultraviolet (UV) radiation from sunlight [20], but is also dependent upon individual factors, including skin pigmentation, predisposition to naevi development and genetic factors. Sunlight UV exposure is high in Australia but varies between States and Territories. Consequently, a comparison of the cancer rates amongst mine/refinery workers was also made with data from WA [21]. Compared with the Australian population, significantly elevated risks were found amongst males and females. However, comparison with the WA population attenuated the risks amongst males, suggesting male workers in mines/refineries are at no greater risk than others living and working in WA. The epidemiology of melanoma differs between males and females: under age 50 years, melanoma is more common among females than males [22]. This has been hypothesized to relate to different sun protection and exposure patterns over the life course, such as intentional and indoor tanning behaviours of younger females [23–26]. However, there is no evidence to suggest female mine/refinery workers would have different tanning behaviours from females in the general population.

The excess of lip cancer (SIR 2.04, 95% CI 1.02–3.65) in the maintenance/production workers working >3 months and <10 years is of interest, particularly given an elevated risk ratio for lip cancer amongst all male workers. Risk factors for lip cancer include age, male gender, poorer socioeconomic circumstances, smoking, alcohol, early life and cumulative sunlight exposure, viral infections and immunosuppression. An association with outdoor work has been previously found [27]. Sun protection practices should continue to be promoted amongst mine/refinery workers.

This linkage revealed a significantly increased incidence of thyroid cancer amongst office workers. Bauxite ore dust is a source of ionizing radiation which is associated with the risk of thyroid cancer; however, radiological assessment at these sites found incremental composite doses of gamma, radon progeny and gross alpha rays within the public exposure limit of 1.0 mSv per year above background [28]. Also, an investigation of bauxite in Guinean and Indian mines did not indicate excessive external gamma radiation doses [29]. Given the excess risk in office workers, it is also unlikely the putative exposure is bauxite ore dust. Thyroid cancer rates can vary according to geographic regions [30,31]; hence the data were further analysed using State-based data, but the increased risk remained. The most reproducible epidemiologically demonstrated causal risk factor for thyroid cancer is childhood exposure to ionizing radiation, but other risk factors are thought to include diet, obesity and endocrine factors. Environmental factors have been implicated in the increasing global rates of thyroid cancer, although there is no conclusive evidence about causation [32].

A new finding since the last linkage was the significantly higher prostate cancer incidence amongst mine/refinery workers who were ‘ever office’ workers [10]. Due to variability in prostate screening access, comparison of incidence rates of prostate cancer is complex (prostate-specific antigen testing was introduced to Australia in the mid-1990s, without standardized screening criteria or programmes). It is possible the increased incidence could be due to screening access, although workplace screening or health promotion is unlikely to differ between office, production or maintenance workers. Educational attainment and socioeconomic status, two factors that are recognized to influence requesting for prostate cancer screening [33], may at least partly explain this finding in office workers.

It is a strength of this study that there is a high level of completeness of the cancer incidence and causes of death data, which are collected by AIHW through a mandatory reporting system. The study also benefits from complete access to employee records allowing detailed extraction of a full job history for every cohort member. However, our findings need to be considered alongside some limitations. First, workers in mines/refineries are active workers and therefore ‘healthier’ than the background population. The relative impact of this healthy worker bias will differ somewhat by disease, with less impact on conditions with mortality after a short period of onset and death (such as some cancers) [34], but a more overt effect on conditions with a more prolonged progression of disability [35]. A further limitation is the cohort size which, although large for this industry, may be insufficient to detect small increases in rarer cancers and causes of death. Additionally, the low number of female workers limited analyses. Finally, assessment of exposures amongst workers was not undertaken at a personal exposure level, or quantitively, and therefore is based on job title ‘ever’. Each person-year at risk within an individual’s job history is separated into the appropriate category but some workers contribute person-years in more than one category.

In summary, a strong healthy worker effect was evident in bauxite mine and alumina refinery workers with all mortality causes, other than mesothelioma, below or similar to expected rates; mesothelioma incidence was significantly higher. There was an excess of thyroid, lip, and prostate cancer cases in some subgroups of male workers, and melanoma cases in female workers, that cannot currently be explained by workplace exposure; this requires further investigation.

**Key learning points**

**What is already known about this subject:**

- The International Agency for Research on Cancer has reported that aluminium production is carcinogenic.
- Excess risks of bladder cancer, lung cancer and cancers at other work sites have been reported.

**What this study adds:**

- Mesothelioma incidence is significantly higher among bauxite mine and alumina refinery workers, partly due to exposures at alumina refineries, and possibly through domestic and previous occupational exposures.
- There is an excess of thyroid, lip and prostate cancer cases in male workers, and melanoma cases in female workers, which cannot currently be explained by workplace exposure.
- There is a healthy worker effect with workers in the bauxite mines and alumina refineries, with an overall lower risk of death from all causes, all malignancies and a range of specific causes.

**What impact this study has on policy or practice:**

- Given the higher rates of melanoma and lip cancer, ongoing optimal sun protection measures are recommended.
- Further investigation needs to be conducted to determine possible causes of excess lip, thyroid and prostate cancer in male workers in bauxite mines and alumina refineries.

## Supplementary Material

kqae069_suppl_Supplementary_Tables

## Data Availability

Linkage data were obtained from the Australian Institute for Health and Welfare and permission would need to be obtained from them for additional data usage.
