# Supplementary material for: Bauxite mine and alumina refinery workers: mortality and cancer risk
Source: Occup Med (Lond). 2024 Sep 11;74(7):508–13. doi: 10.1093/occmed/kqae069 (PMC11444375; doi:10.1093/occmed/kqae069)
Supplement: kqae069_suppl_Supplementary_Tables [file kqae069_suppl_supplementary_tables.docx]

**Table 1(a)** **Comparison of mortality rates for major and specific causes of 6207 eligible male mine/refinery workers, to the Australian general population**

|  | **All Mine/Refinery (N=6,207 )** | | | **Ever Production (N=3,021)** | | | **Ever Maintenance (N=3,368)** | | | **Ever Office (N=1,725)** | | |
| --- | --- | --- | --- | --- | --- | --- | --- | --- | --- | --- | --- | --- |
| **Cause of death categories+** | **Obs** | **Exp** | **SMR (95% CI)** | **Obs** | **Exp** | **SMR (95% CI)** | **Obs** | **Exp** | **SMR (95% CI)** | **Obs** | **Exp** | **SMR (95% CI)** |
| **All Malignancies** | 352 | 397.42 | 0.89 (0.80 - 0.98) | 182 | 191.21 | 0.95 (0.82 - 1.10) | 161 | 187.26 | 0.86 (0.73 - 1.00) | 87 | 106.17 | 0.82 (0.66 - 1.01) |
| Oesophagus | 12 | 15.20 | 0.79 (0.41 - 1.38) | 7 | 7.45 | 0.94 (0.38 - 1.94) | 7 | 7.25 | 0.97 (0.39 - 1.99) | 4 | 4.13 | 0.97 (0.26 - 2.48) |
| Stomach | 14 | 13.58 | 1.03 (0.56 - 1.73) | 9 | 6.50 | 1.38 (0.63 - 2.63) | 7 | 6.42 | 1.09 (0.44 - 2.25) | 3 | 3.61 | 0.83 (0.17 - 2.43) |
| Colorectal | 41 | 43.96 | 0.93 (0.67 - 1.27) | 19 | 21.13 | 0.90 (0.54 - 1.40) | 16 | 20.68 | 0.77 (0.44 - 1.26) | 13 | 11.71 | 1.11 (0.59 - 1.90) |
| Liver | 11 | 14.64 | 0.75 (0.38 - 1.34) | 6 | 7.26 | 0.83 (0.30 - 1.80) | 5 | 7.06 | 0.71 (0.23 - 1.65) | <3 | 4.04 | 0.50 (0.06 - 1.79) |
| Pancreas | 15 | 20.45 | 0.73 (0.41 - 1.21) | 7 | 9.99 | 0.70 (0.28 - 1.44) | 8 | 9.72 | 0.82 (0.36 - 1.62) | 3 | 5.54 | 0.54 (0.11 - 1.58) |
| Lung | 85 | 91.12 | 0.93 (0.75 - 1.15) | 47 | 43.73 | 1.07 (0.79 - 1.43) | 34 | 42.75 | 0.80 (0.55 - 1.11) | 18 | 24.24 | 0.74 (0.44 - 1.17) |
| Mesothelioma, pleural | 17 | 7.60 | 2.24 (1.30 - 3.58) | 9 | 3.72 | 2.42 (1.11 - 4.60) | 8 | 3.53 | 2.27 (0.98 - 4.47) | 5 | 2.05 | 2.44 (0.79 - 5.70) |
| Melanoma | 17 | 16.71 | 1.02 (0.59 - 1.63) | 7 | 8.14 | 0.86 (0.35 - 1.77) | 9 | 7.99 | 1.13 (0.51 - 2.14) | 6 | 4.50 | 1.33 (0.49 - 2.90) |
| Prostate | 27 | 34.86 | 0.77 (0.51 - 1.13) | 16 | 16.20 | 0.99 (0.56 - 1.60) | 11 | 15.93 | 0.69 (0.34 - 1.24) | 7 | 9.12 | 0.77 (0.31 - 1.58) |
| Bladder | 12 | 9.08 | 1.32 (0.68 - 2.31) | 5 | 4.28 | 1.17 (0.38 - 2.73) | 5 | 4.18 | 1.20 (0.39 - 2.79) | 5 | 2.39 | 2.09 (0.68 - 4.88) |
| Kidney | 6 | 10.09 | 0.59 (0.22 - 1.29) | <3 | 4.92 | 0.20 (0.01 - 1.13) | 3 | 4.80 | 0.62 (0.13 - 1.83) | <3 | 2.73 | 0.73 (0.09 - 2.65) |
| Brain | 12 | 15.54 | 0.77 (0.40 - 1.35) | 8 | 7.62 | 1.05 (0.45 - 2.07) | 5 | 7.59 | 0.66 (0.21 - 1.54) | 0 | 4.22 | - |
| **All Metabolic** | 20 | 39.29 | 0.51 (0.31 - 0.79) | 5 | 18.79 | 0.27 (0.09 - 0.62) | 13 | 18.43 | 0.71 (0.38 - 1.21) | 4 | 10.46 | 0.38 (0.10 - 0.98) |
| **All Mental And Behavioural** | 11 | 26.68 | 0.41 (0.21 - 0.74) | 5 | 12.30 | 0.41 (0.13 - 0.95) | 5 | 12.41 | 0.40 (0.13 - 0.94) | <3 | 6.89 | 0.29 (0.04 - 1.05) |
| **All Nervous System** | 31 | 34.29 | 0.90 (0.61 - 1.28) | 10 | 16.27 | 0.61 (0.29 - 1.13) | 20 | 16.08 | 1.24 (0.76 - 1.92) | 8 | 9.08 | 0.88 (0.38 - 1.74) |
| Alzheimer's Disease | 9 | 7.06 | 1.27 (0.58 - 2.42) | 3 | 3.25 | 0.92 (0.19 - 2.70) | 5 | 3.11 | 1.61 (0.52 - 3.76) | <3 | 1.83 | 1.09 (0.13 - 3.95) |
| Parkinson's Disease | 9 | 7.28 | 1.24 (0.57 - 2.35) | <3 | 3.38 | 0.30 (0.01 - 1.65) | 6 | 3.23 | 1.86 (0.68 - 4.04) | 3 | 1.89 | 1.58 (0.33 - 4.63) |
| **All Circulatory** | 251 | 321.85 | 0.78 (0.69 - 0.88) | 104 | 149.69 | 0.69 (0.57 - 0.84) | 124 | 150.49 | 0.82 (0.69 - 0.98) | 63 | 84.07 | 0.75 (0.58 - 0.96) |
| Ischaemic Heart Disease | 163 | 197.48 | 0.83 (0.70 - 0.96) | 70 | 92.02 | 0.76 (0.59 - 0.96) | 77 | 92.56 | 0.83 (0.66 - 1.04) | 41 | 51.58 | 0.79 (0.57 - 1.08) |
| Cerebrovascular | 35 | 53.43 | 0.66 (0.46 - 0.91) | 14 | 24.53 | 0.57 (0.31 - 0.96) | 19 | 24.74 | 0.77 (0.46 - 1.20) | 9 | 13.88 | 0.65 (0.30 - 1.23) |
| Other Heart Disease | 34 | 50.25 | 0.68 (0.47 - 0.95) | 14 | 23.62 | 0.59 (0.32 - 0.99) | 20 | 23.49 | 0.85 (0.52 - 1.31) | 6 | 13.22 | 0.45 (0.17 - 0.99) |
| **All Respiratory** | 49 | 77.15 | 0.64 (0.47 - 0.84) | 19 | 35.79 | 0.53 (0.32 - 0.83) | 24 | 35.63 | 0.67 (0.43 - 1.00) | 13 | 20.12 | 0.65 (0.34 - 1.10) |
| COPD | 19 | 43.37 | 0.44 (0.26 - 0.68) | 8 | 20.07 | 0.40 (0.17 - 0.79) | 9 | 20.00 | 0.45 (0.21 - 0.85) | 4 | 11.30 | 0.35 (0.10 - 0.91) |
| Asthma | <3 | 3.46 | 0.29 (0.01 - 1.61) | 0 | 1.58 | - | 0 | 1.67 | - | <3 | 0.88 | 1.14 (0.03 - 6.36) |
| Asbestosis | <3 | 1.06 | 1.88 (0.23 - 6.80) | <3 | 0.50 | 2.01 (0.05 - 11.20) | <3 | 0.47 | 2.12 (0.05 - 11.79) | 0 | 0.28 | - |
| **All Digestive** | 17 | 44.10 | 0.39 (0.22 - 0.62) | 9 | 21.33 | 0.42 (0.19 - 0.80) | 12 | 21.08 | 0.57 (0.29 - 0.99) | 3 | 11.85 | 0.25 (0.05 - 0.74) |
| Liver Disease | 7 | 26.0291 | 0.27 (0.11 - 0.55) | 4 | 12.86 | 0.31 (0.08 - 0.80) | 6 | 12.68 | 0.47 (0.17 - 1.03) | <3 | 7.09 | 0.14 (0.00 - 0.79) |
| **All Urinary** | 6 | 14.09 | 0.43 (0.16 - 0.93) | 5 | 6.48 | 0.77 (0.25 - 1.80) | <3 | 6.45 | 0.16 (0.00 - 0.86) | 4 | 3.68 | 1.09 (0.30 - 2.78) |
| **All Injury And Trauma** | 82 | 118.95 | 0.69 (0.55 - 0.86) | 43 | 53.73 | 0.80 (0.58 - 1.08) | 40 | 61.81 | 0.65 (0.46 - 0.88) | 17 | 30.15 | 0.56 (0.33 - 0.90) |
| Accidents | 45 | 66.17 | 0.68 (0.50 - 0.91) | 27 | 29.59 | 0.91 (0.60 - 1.33) | 17 | 34.22 | 0.50 (0.29 - 0.80) | 8 | 16.63 | 0.48 (0.21 - 0.95) |
| Suicide | 34 | 44.16 | 0.77 (0.53 - 1.08) | 15 | 20.19 | 0.74 (0.42 - 1.23) | 21 | 23.13 | 0.91 (0.56 - 1.39) | 9 | 11.30 | 0.80 (0.36 - 1.51) |
| **All Other Causes** | 20 | 42.12 | 0.47 (0.29 - 0.73) | 12 | 20.14 | 0.60 (0.31 - 1.04) | 8 | 20.40 | 0.39 (0.17 - 0.77) | 3 | 11.22 | 0.27 (0.06 - 0.78) |
| **All Death Causes Combined*** | 842 | 1115.94 | 0.75 (0.70 - 0.81) | 394 | 525.73 | 0.75 (0.68 - 0.83) | 408 | 530.03 | 0.77 (0.70 - 0.85) | 207 | 293.68 | 0.70 (0.61 - 0.81) |

* 3 deaths included in all deaths which have no cause of death coded. COPD = Chronic Obstructive Pulmonary Disease.
+ Refer to Table S1 for ICD-9/ICD-10 definitions of death categories. Sum of number of deaths in the 3 work categories does not equal number of deaths in all mine/refinery group, as ever work classification is not mutually exclusive. In addition, some job histories were missing; consequently, some deaths will not appear in the classification into work categories.

**Table 1(b)** **Comparison of mortality rates of 728 eligible female mine/refinery workers, to the Australian general population**

|  | **All Mine/Refinery (N=728)** | | | **Ever Production (N=143)** | | | **Ever Maintenance (N=131)** | | | **Ever Office (N=602)** | | |
| --- | --- | --- | --- | --- | --- | --- | --- | --- | --- | --- | --- | --- |
| **Cause of death categories+** | **Obs** | **Exp** | **SMR (95% CI)** | **Obs** | **Exp** | **SMR (95% CI)** | **Obs** | **Exp** | **SMR (95% CI)** | **Obs** | **Exp** | **SMR (95% CI)** |
| **All Malignancies** | 23 | 23.11 | 1.00 (0.63 - 1.49) | <3 | 3.70 | 0.54 (0.07 - 1.95) | 3 | 4.03 | 0.74 (0.15 - 2.18) | 20 | 18.83 | 1.06 (0.65 - 1.64) |
| **All Metabolic** | 0 | 1.92 | - | 0 | 0.28 | - | 0 | 0.32 | - | 0 | 1.57 | - |
| **All Mental And Behavioural** | <3 | 1.41 | 0.71 (0.02 - 3.94) | 0 | 0.15 | - | 0 | 0.23 | - | <3 | 1.16 | 0.87 (0.02 - 4.82) |
| **All Nervous System** | <3 | 2.10 | 0.95 (0.12 - 3.44) | 0 | 0.30 | - | <3 | 0.35 | 2.88 (0.07 - 16.05) | <3 | 1.71 | 0.58 (0.01 - 3.25) |
| **All Circulatory** | 9 | 11.17 | 0.81 (0.37 - 1.53) | <3 | 1.44 | 0.70 (0.02 - 3.88) | <3 | 1.83 | 1.10 (0.13 - 3.96) | 8 | 9.14 | 0.87 (0.38 - 1.72) |
| **All Respiratory** | 0 | 3.66 | - | 0 | 0.52 | - | 0 | 0.62 | - | 0 | 2.97 | - |
| **All Digestive** | 0 | 1.97 | - | 0 | 0.30 | - | 0 | 0.33 | - | 0 | 1.61 | - |
| **All Urinary** | 0 | 0.80 | - | 0 | 0.10 | - | 0 | 0.13 | - | 0 | 0.66 | - |
| **All Injury And Trauma** | 3 | 4.43 | 0.68 (0.14 - 1.98) | <3 | 0.69 | 1.45 (0.04 - 8.07) | 0 | 0.72 | - | <3 | 3.66 | 0.55 (0.07 - 1.98) |
| **All Other Causes** | 0 | 4.69 | - | 0 | 0.63 | - | 0 | 0.77 | - | 0 | 3.84 | - |
| **All Death Causes Combined** | 38 | 53.04 | 0.72 (0.51 - 0.98) | 4 | 7.85 | 0.51 (0.14 - 1.30) | 6 | 8.97 | 0.67 (0.25 - 1.46) | 32 | 43.33 | 0.74 (0.51 - 1.04) |

+ The sum of causes of death in the 3 work categories does not always equal the number of deaths in the all mine/refinery group, as the ever worked classification is not mutually exclusive. In addition, some job histories were missing; consequently, some deaths do not appear in the classification into work categories

**Table 2** **Comparison of mortality rates for major and specific causes amongst 6207 eligible male mine/refinery workers, to the Australian general population presented by duration of employment in maintenance/production**

|  | **No EMPLOYMENT IN PRODUCTION OR MAINTENANCE** | | | **DURATION OF EMPLOYMENT IN PRODUCTION OR MAINTENANCE** | | | | | | | | |
| --- | --- | --- | --- | --- | --- | --- | --- | --- | --- | --- | --- | --- |
|  |  |  |  | **3 months to < 10 years** | | | **10- 20 years** | | | **> 20 years** | | |
| **Cause of death categories** | **Obs** | **Exp** | **SMR (95% CI)** | **Obs** | **Exp** | **SMR (95% CI)** | **Obs** | **Exp** | **SMR (95% CI)** | **Obs** | **Exp** | **SMR (95% CI)** |
| **All Malignancies** | 48 | 70.06 | 0.69 (0.51 - 0.91) | 91 | 84.54 | 1.08 (0.87 - 1.32) | 125 | 123.65 | 1.01 (0.84 - 1.20) | 81 | 116.14 | 0.70 (0.55 - 0.87) |
| Oesophagus | <3 | 2.61 | 0.38 (0.01 - 2.13) | 5 | 3.32 | 1.50 (0.49 - 3.51) | <3 | 4.45 | 0.45 (0.05 - 1.62) | 4 | 4.70 | 0.85 (0.23 - 2.18) |
| Stomach | <3 | 2.39 | 0.84 (0.10 - 3.02) | <3 | 2.98 | 0.34 (0.01 - 1.87) | 5 | 4.33 | 1.15 (0.37 - 2.70) | 6 | 3.78 | 1.59 (0.58 - 3.46) |
| Colorectal | 8 | 7.73 | 1.04 (0.45 - 2.04) | 7 | 9.31 | 0.75 (0.30 - 1.55) | 15 | 13.97 | 1.07 (0.60 - 1.77) | 9 | 12.61 | 0.71 (0.33 - 1.35) |
| Liver | <3 | 2.48 | 0.40 (0.01 - 2.24) | 3 | 3.37 | 0.89 (0.18 - 2.60) | 6 | 3.98 | 1.51 (0.55 - 3.28) | <3 | 4.70 | 0.21 (0.01 - 1.19) |
| Pancreas | <3 | 3.54 | 0.28 (0.01 - 1.58) | 5 | 4.39 | 1.14 (0.37 - 2.66) | 6 | 6.02 | 1.00 (0.37 - 2.17) | 3 | 6.35 | 0.47 (0.10 - 1.38) |
| Lung | 12 | 16.09 | 0.75 (0.39 - 1.30) | 20 | 18.32 | 1.09 (0.67 - 1.69) | 33 | 29.02 | 1.14 (0.78 - 1.60) | 19 | 26.99 | 0.70 (0.42 - 1.10) |
| Mesothelioma, pleural | <3 | 1.34 | 0.75 (0.02 - 4.16) | 7 | 1.47 | 4.75 (1.91 - 9.78) | 4 | 2.27 | 1.76 (0.48 - 4.50) | 4 | 2.46 | 1.63 (0.44 - 4.17) |
| Melanoma | 4 | 2.87 | 1.40 (0.38 - 3.57) | 3 | 4.25 | 0.71 (0.15 - 2.06) | 6 | 4.90 | 1.22 (0.45 - 2.67) | 4 | 4.57 | 0.87 (0.24 - 2.24) |
| Prostate | <3 | 6.59 | 0.30 (0.04 - 1.10) | 6 | 5.67 | 1.06 (0.39 - 2.30) | 11 | 12.05 | 0.91 (0.46 - 1.63) | 6 | 10.28 | 0.58 (0.21 - 1.27) |
| Bladder | <3 | 1.68 | 1.19 (0.14 - 4.31) | 5 | 1.63 | 3.06 (0.99 - 7.14) | 3 | 3.04 | 0.99 (0.20 - 2.89) | <3 | 2.66 | 0.75 (0.09 - 2.71) |
| Kidney | <3 | 1.74 | 1.15 (0.14 - 4.14) | <3 | 2.26 | 0.44 (0.01 - 2.46) | <3 | 3.04 | 0.66 (0.08 - 2.38) | <3 | 2.97 | 0.34 (0.01 - 1.88) |
| Brain | 0 | 2.59 |  | <3 | 4.24 | 0.47 (0.06 - 1.70) | 7 | 4.36 | 1.60 (0.65 - 3.31) | 3 | 4.23 | 0.71 (0.15 - 2.07) |
| **All Metabolic** | 3 | 7.04 | 0.43 (0.09 - 1.24) | 5 | 8.18 | 0.61 (0.20 - 1.43) | 7 | 12.33 | 0.57 (0.23 - 1.17) | 5 | 11.44 | 0.44 (0.14 - 1.02) |
| Diabetes | 3 | 5.16 | 0.58 (0.12 - 1.70) | 5 | 5.71 | 0.88 (0.28 - 2.04) | 6 | 9.08 | 0.66 (0.24 - 1.44) | 5 | 8.44 | 0.59 (0.19 - 1.38) |
| **All Mental And Behavioural** | <3 | 5.01 | 0.40 (0.05 - 1.44) | <3 | 6.77 | 0.30 (0.04 - 1.07) | 4 | 8.53 | 0.47 (0.13 - 1.20) | 3 | 6.16 | 0.49 (0.10 - 1.42) |
| Dementia | <3 | 3.05 | 0.33 (0.01 - 1.83) | 0 | 2.12 |  | <3 | 5.54 | 0.36 (0.04 - 1.30) | 3 | 4.21 | 0.71 (0.15 - 2.08) |
| **All Nervous System** | 5 | 6.21 | 0.81 (0.26 - 1.88) | 7 | 7.54 | 0.93 (0.37 - 1.91) | 12 | 10.66 | 1.13 (0.58 - 1.97) | 7 | 9.62 | 0.73 (0.29 - 1.50) |
| Alzheimer's Disease | <3 | 1.41 | 1.42 (0.17 - 5.12) | <3 | 1.03 | 1.95 (0.24 - 7.03) | <3 | 2.58 | 0.78 (0.09 - 2.80) | 3 | 1.99 | 1.51 (0.31 - 4.40) |
| Parkinson's Disease | <3 | 1.42 | 1.41 (0.17 - 5.09) | <3 | 1.10 | 0.91 (0.02 - 5.07) | 5 | 2.56 | 1.95 (0.63 - 4.56) | <3 | 2.15 | 0.47 (0.01 - 2.59) |
| **All Circulatory** | 48 | 58.96 | 0.81 (0.60 - 1.08) | 60 | 65.59 | 0.91 (0.70 - 1.18) | 90 | 111.59 | 0.81 (0.65 - 0.99) | 50 | 83.18 | 0.60 (0.45 - 0.79) |
| IHD | 31 | 35.88 | 0.86 (0.59 - 1.23) | 43 | 40.89 | 1.05 (0.76 - 1.42) | 55 | 68.77 | 0.80 (0.60 - 1.04) | 32 | 50.37 | 0.64 (0.43 - 0.90) |
| Cerebrovascular | 6 | 10.05 | 0.60 (0.22 - 1.30) | 3 | 10.08 | 0.30 (0.06 - 0.87) | 18 | 19.00 | 0.95 (0.56 - 1.50) | 8 | 13.88 | 0.58 (0.25 - 1.14) |
| Other Heart Disease | 5 | 9.19 | 0.54 (0.18 - 1.27) | 9 | 10.73 | 0.84 (0.38 - 1.59) | 13 | 16.60 | 0.78 (0.42 - 1.34) | 6 | 13.36 | 0.45 (0.16 - 0.98) |
| **All Respiratory** | 9 | 14.41 | 0.62 (0.29 - 1.19) | 7 | 14.10 | 0.50 (0.20 - 1.02) | 19 | 26.54 | 0.72 (0.43 - 1.12) | 14 | 21.50 | 0.65 (0.36 - 1.09) |
| COPD | 3 | 8.11 | 0.37 (0.08 - 1.08) | 4 | 7.33 | 0.55 (0.15 - 1.40) | 9 | 15.11 | 0.60 (0.27 - 1.13) | 3 | 12.49 | 0.24 (0.05 - 0.70) |
| Asthma | <3 | 0.60 | 1.66 (0.04 - 9.25) | 0 | 1.05 |  | 0 | 1.13 |  | 0 | 0.65 |  |
| Asbestosis | 0 | 0.20 |  | 0 | 0.16 |  | <3 | 0.37 | 2.71 (0.07 - 15.08) | <3 | 0.32 | 3.14 (0.08 - 17.52) |
| **All Digestive** | <3 | 7.66 | 0.26 (0.03 - 0.94) | 4 | 10.84 | 0.37 (0.10 - 0.94) | 9 | 13.54 | 0.66 (0.30 - 1.26) | <3 | 11.72 | 0.17 (0.02 - 0.62) |
| Liver Disease | 0 | 4.32 |  | <3 | 7.23 | 0.28 (0.03 - 1.00) | 5 | 7.46 | 0.67 (0.22 - 1.57) | 0 | 6.82 |  |
| **All Urinary** | <3 | 2.71 | 0.37 (0.01 - 2.06) | 3 | 2.47 | 1.21 (0.25 - 3.55) | 0 | 4.96 |  | <3 | 3.85 | 0.52 (0.06 - 1.88) |
| Kidney Failure | <3 | 1.80 | 0.56 (0.01 - 3.10) | <3 | 1.55 | 1.29 (0.16 - 4.66) | 0 | 3.27 |  | <3 | 2.60 | 0.77 (0.09 - 2.78) |
| **All Injury And Trauma** | 8 | 19.40 | 0.41 (0.18 - 0.81) | 42 | 50.77 | 0.83 (0.60 - 1.12) | 23 | 29.55 | 0.78 (0.49 - 1.17) | 8 | 18.39 | 0.44 (0.19 - 0.86) |
| Accidents | 5 | 10.91 | 0.46 (0.15 - 1.07) | 27 | 27.88 | 0.97 (0.64 - 1.41) | 6 | 16.51 | 0.36 (0.13 - 0.79) | 6 | 10.40 | 0.58 (0.21 - 1.26) |
| Suicide | 3 | 7.09 | 0.42 (0.09 - 1.24) | 15 | 19.29 | 0.78 (0.44 - 1.28) | 14 | 10.89 | 1.29 (0.70 - 2.16) | <3 | 6.58 | 0.30 (0.04 - 1.10) |
| **All Other Causes** | 3 | 7.31 | 0.41 (0.08 - 1.20) | 3 | 11.96 | 0.25 (0.05 - 0.73) | 9 | 12.24 | 0.74 (0.34 - 1.40) | 5 | 10.30 | 0.49 (0.16 - 1.13) |
| **All Death Causes Combined*** | 132 | 198.77 | 0.66 (0.56 - 0.79) | 224 | 262.77 | 0.85 (0.74 - 0.97) | 298 | 353.61 | 0.84 (0.75 - 0.94) | 177 | 292.30 | 0.61 (0.52 - 0.70) |

* 3 deaths included in all deaths which have no cause of death coded. COPD = Chronic Obstructive Pulmonary Disease.

+ The sum of causes of death in the 3 work categories does not always equal the number of deaths in the all mine/refinery group, as the ever worked classification is not mutually exclusive. In addition, some job histories were missing; consequently, some deaths do not appear in the classification into work categories

**Table 3 (a) Comparison of cancer incidence rates amongst 6207 eligible male mine/refinery workers, to the Australian general population presented by duration of employment in maintenance/production**

|  | **All Mine/Refinery (N=6,207)** | | | **Ever Production (N=3,024)** | | | **Ever Maintenance (N=3,368)** | | | **Ever Office (N=1,725)** | | |
| --- | --- | --- | --- | --- | --- | --- | --- | --- | --- | --- | --- | --- |
| **Cancer categories**+ | **O** | **E** | **SIR (95% CI)** | **O** | **E** | **SIR (95% CI)** | **O** | **E** | **SIR (95% CI)** | **O** | **E** | **SIR (95% CI)** |
| **Lip, Oral Cavity And Pharynx** | 48 | 53.91 | 0.89 (0.66 - 1.18) | 31 | 26.58 | 1.17 (0.79 - 1.66) | 28 | 26.51 | 1.06 (0.70 - 1.53) | 8 | 14.72 | 0.54 (0.23 - 1.07) |
| Lip | 24 | 17.56 | 1.37 (0.88 - 2.03) | 14 | 8.49 | 1.65 (0.90 - 2.77) | 12 | 8.66 | 1.39 (0.72 - 2.42) | 6 | 4.70 | 1.28 (0.47 - 2.78) |
| Pharynx | 9 | 15.43 | 0.58 (0.27 - 1.11) | 6 | 7.69 | 0.78 (0.29 - 1.70) | 6 | 7.61 | 0.79 (0.29 - 1.72) | 0 | 4.27 | - |
| **Digestive Organs** | 222 | 248.67 | 0.89 (0.78 - 1.02) | 106 | 122.05 | 0.87 (0.71 - 1.05) | 109 | 119.08 | 0.92 (0.75 - 1.10) | 60 | 67.61 | 0.89 (0.68 - 1.14) |
| Oesophagus | 18 | 17.23 | 1.04 (0.62 - 1.65) | 12 | 8.50 | 1.41 (0.73 - 2.47) | 9 | 8.29 | 1.09 (0.50 - 2.06) | 5 | 4.72 | 1.06 (0.34 - 2.47) |
| Stomach | 22 | 26.01 | 0.85 (0.53 - 1.28) | 12 | 12.62 | 0.95 (0.49 - 1.66) | 12 | 12.40 | 0.97 (0.50 - 1.69) | 6 | 7.00 | 0.86 (0.31 - 1.87) |
| Colorectal | 140 | 152.71 | 0.92 (0.77 - 1.08) | 63 | 74.84 | 0.84 (0.65 - 1.08) | 65 | 72.92 | 0.89 (0.69 - 1.14) | 40 | 41.40 | 0.97 (0.69 - 1.32) |
| Colon | 71 | 88.87 | 0.80 (0.62 - 1.01) | 32 | 43.41 | 0.74 (0.50 - 1.04) | 32 | 42.28 | 0.76 (0.52 - 1.07) | 20 | 23.99 | 0.83 (0.51 - 1.29) |
| Rectum | 69 | 59.58 | 1.16 (0.90 - 1.47) | 31 | 29.38 | 1.06 (0.72 - 1.50) | 33 | 28.68 | 1.15 (0.79 - 1.62) | 20 | 16.24 | 1.23 (0.75 - 1.90) |
| Liver | 13 | 18.55 | 0.70 (0.37 - 1.20) | 6 | 9.29 | 0.65 (0.24 - 1.41) | 7 | 9.10 | 0.77 (0.31 - 1.58) | 4 | 5.17 | 0.77 (0.21 - 1.98) |
| Gallbladder | 7 | 5.76 | 1.21 (0.49 - 2.50) | 4 | 2.81 | 1.42 (0.39 - 3.64) | 3 | 2.74 | 1.10 (0.23 - 3.20) | <3 | 1.56 | 0.64 (0.02 - 3.58) |
| Pancreas | 18 | 23.76 | 0.76 (0.45 - 1.20) | 7 | 11.67 | 0.60 (0.24 - 1.24) | 12 | 11.35 | 1.06 (0.55 - 1.85) | 3 | 6.48 | 0.46 (0.10 - 1.35) |
| **Respiratory & Intrathoracic Organs** | 108 | 135.89 | 0.79 (0.65 - 0.96) | 59 | 65.87 | 0.90 (0.68 - 1.16) | 50 | 64.24 | 0.78 (0.58 - 1.03) | 20 | 36.45 | 0.55 (0.34 - 0.85) |
| Larynx | 7 | 12.47 | 0.56 (0.23 - 1.16) | <3 | 6.11 | 0.33 (0.04 - 1.18) | 4 | 5.93 | 0.67 (0.18 - 1.73) | <3 | 3.37 | 0.59 (0.07 - 2.15) |
| Lung | 96 | 120.01 | 0.80 (0.65 - 0.98) | 54 | 58.10 | 0.93 (0.70 - 1.21) | 45 | 56.63 | 0.79 (0.58 - 1.06) | 17 | 32.17 | 0.53 (0.31 - 0.85) |
| **Melanoma** | 164 | 134.07 | 1.22 (1.04 - 1.43) | 73 | 65.86 | 1.11 (0.87 - 1.39) | 83 | 65.95 | 1.26 (1.00 - 1.56) | 42 | 36.48 | 1.15 (0.83 - 1.56) |
| **Mesothelioma** | 24 | 9.61 | 2.50 (1.60 - 3.71) | 11 | 4.69 | 2.34 (1.17 - 4.19) | 12 | 4.49 | 2.68 (1.38 - 4.67) | 6 | 2.58 | 2.32 (0.85 - 5.05) |
| **Male Reproductive Organs** | 357 | 334.56 | 1.07 (0.96 - 1.18) | 175 | 166.51 | 1.05 (0.90 - 1.22) | 165 | 162.59 | 1.01 (0.87 - 1.18) | 111 | 92.57 | 1.20 (0.99 - 1.44) |
| Prostate | 346 | 302.65 | 1.14 (1.03 - 1.27) | 171 | 152.26 | 1.12 (0.96 - 1.30) | 157 | 147.11 | 1.07 (0.91 - 1.25) | 108 | 83.29 | 1.30 (1.06 - 1.57) |
| Testis | 9 | 13.05 | 0.69 (0.32 - 1.31) | 4 | 5.76 | 0.69 (0.19 - 1.78) | 7 | 7.09 | 0.99 (0.40 - 2.04) | <3 | 3.24 | 0.62 (0.07 - 2.23) |
| **Urinary Tract** | 70 | 70.13 | 1.00 (0.78 - 1.26) | 33 | 34.24 | 0.96 (0.66 - 1.35) | 34 | 33.65 | 1.01 (0.70 - 1.41) | 23 | 19.01 | 1.21 (0.77 - 1.82) |
| Kidney | 31 | 34.35 | 0.90 (0.61 - 1.28) | 16 | 17.05 | 0.94 (0.54 - 1.52) | 17 | 16.86 | 1.01 (0.59 - 1.61) | 10 | 9.48 | 1.05 (0.51 - 1.94) |
| Bladder | 36 | 31.21 | 1.15 (0.81 - 1.60) | 15 | 14.97 | 1.00 (0.56 - 1.65) | 16 | 14.66 | 1.09 (0.62 - 1.77) | 12 | 8.29 | 1.45 (0.75 - 2.53) |
| **Brain And Other CNS** | 17 | 19.26 | 0.88 (0.51 - 1.41) | 12 | 9.38 | 1.28 (0.66 - 2.23) | 6 | 9.52 | 0.63 (0.23 - 1.37) | <3 | 5.20 | 0.19 (0.00 - 1.07) |
| Brain | 17 | 18.52 | 0.92 (0.53 - 1.47) | 12 | 9.02 | 1.33 (0.69 - 2.32) | 6 | 9.14 | 0.66 (0.24 - 1.43) | <3 | 5.00 | 0.20 (0.01 - 1.11) |
| **Thyroid & Other Endocrine Glands** | 21 | 10.56 | 1.99 (1.23 - 3.04) | 8 | 5.17 | 1.55 (0.67 - 3.05) | 6 | 5.43 | 1.11 (0.41 - 2.41) | 10 | 2.88 | 3.47 (1.66 - 6.38) |
| Thyroid | 20 | 9.75 | 2.05 (1.25 - 3.17) | 8 | 4.78 | 1.67 (0.72 - 3.30) | 6 | 5.02 | 1.20 (0.44 - 2.60) | 9 | 2.66 | 3.38 (1.54 - 6.41) |
| **Unknown Site** | 19 | 26.84 | 0.71 (0.43 - 1.11) | 7 | 12.86 | 0.54 (0.22 - 1.12) | 9 | 12.66 | 0.71 (0.33 - 1.35) | 5 | 7.15 | 0.70 (0.23 - 1.63) |
| **Lymphoid, Haematopoietic & Related Tissue** | 79 | 102.13 | 0.77 (0.61 - 0.96) | 39 | 49.91 | 0.78 (0.56 - 1.07) | 32 | 49.81 | 0.64 (0.44 - 0.91) | 26 | 27.70 | 0.94 (0.61 - 1.38) |
| Hodgkins | <3 | 5.13 | 0.19 (0.00 - 1.09) | 0 | 2.36 | - | <3 | 2.67 | 0.37 (0.01 - 2.08) | 0 | 1.32 | - |
| Non-Hodgkin Lymphoma | 40 | 48.03 | 0.83 (0.60 - 1.13) | 22 | 23.61 | 0.93 (0.58 - 1.41) | 18 | 23.45 | 0.77 (0.45 - 1.21) | 10 | 13.09 | 0.76 (0.37 - 1.41) |
| Multiple Myeloma | 12 | 15.22 | 0.79 (0.41 - 1.38) | 7 | 7.51 | 0.93 (0.37 - 1.92) | 3 | 7.31 | 0.41 (0.08 - 1.20) | 6 | 4.17 | 1.44 (0.53 - 3.13) |
| Leukaemia | 25 | 32.43 | 0.77 (0.50 - 1.14) | 9 | 15.77 | 0.57 (0.26 - 1.08) | 10 | 15.74 | 0.64 (0.30 - 1.17) | 9 | 8.76 | 1.03 (0.47 - 1.95) |
| **Other Cancers** | 21 | 42.30 | 0.50 (0.31 - 0.76) | 11 | 20.53 | 0.54 (0.27 - 0.96) | 8 | 20.44 | 0.39 (0.17 - 0.77) | 6 | 11.40 | 0.53 (0.19 - 1.15) |
| Connective Tissue | 3 | 7.49 | 0.40 (0.08 - 1.17) | 3 | 3.63 | 0.83 (0.17 - 2.41) | 0 | 3.70 | - | <3 | 2.02 | 0.50 (0.01 - 2.76) |
| Myelodysplastic | 4 | 7.77 | 0.51 (0.14 - 1.32) | <3 | 3.79 | 0.53 (0.06 - 1.91) | <3 | 3.59 | 0.28 (0.01 - 1.55) | <3 | 2.10 | 0.95 (0.12 - 3.44) |
| **All Malignancies** | 1150 | 1187.95 | 0.97 (0.91 - 1.03) | 565 | 583.66 | 0.97 (0.89 - 1.05) | 542 | 574.35 | 0.94 (0.87 - 1.03) | 318 | 323.75 | 0.98 (0.88 - 1.10) |

+ Refer to Table S2 for ICD-10 classification of cancer categories.

**Table 3 (b) Comparison of cancer incidence rates amongst 728 eligible female mine/refinery workers, to the Australian general population presented by duration of employment in maintenance/production**

|  | **All Mine/Refinery (N=728)** | | | **Ever Production (N=143)** | | | **Ever Maintenance (N=131)** | | | **Ever Office (N=602)** | | |
| --- | --- | --- | --- | --- | --- | --- | --- | --- | --- | --- | --- | --- |
| **Cancer categories**+ | **O** | **E** | **SIR (95% CI)** | **O** | **E** | **SIR (95% CI)** | **O** | **E** | **SIR (95% CI)** | **O** | **E** | **SIR (95% CI)** |
| **Lip, Oral Cavity And Pharynx** | <3 | 1.69 | 1.18 (0.14 - 4.28) | <3 | 0.28 | 3.52 (0.09 - 19.60) | 0 | 0.29 | - | <3 | 1.39 | 0.72 (0.02 - 4.02) |
| **Digestive Organs** | 15 | 13.45 | 1.11 (0.62 - 1.84) | <3 | 2.21 | 0.45 (0.01 - 2.52) | <3 | 2.36 | 0.42 (0.01 - 2.36) | 14 | 10.96 | 1.28 (0.70 - 2.14) |
| **Respiratory And Intrathoracic Organs** | 4 | 5.95 | 0.67 (0.18 - 1.72) | <3 | 0.98 | 2.03 (0.25 - 7.34) | 0 | 1.07 | - | 4 | 4.84 | 0.83 (0.23 - 2.12) |
| **Melanoma** | 20 | 10.17 | 1.97 (1.20 - 3.04) | 5 | 1.70 | 2.93 (0.95 - 6.84) | <3 | 1.73 | 0.58 (0.01 - 3.22) | 19 | 8.38 | 2.27 (1.36 - 3.54) |
| **Mesothelioma** | <3 | 0.16 | 12.73 (1.54 - 45.98) | <3 | 0.03 | 38.93 (0.99 - 16.92) | <3 | 0.03 | 35.58 (0.90 - 198.25) | <3 | 0.13 | 7.83 (0.20 - 43.61) |
| **Breast** | 29 | 30.58 | 0.95 (0.64 - 1.36) | 4 | 5.26 | 0.76 (0.21 - 1.95) | 7 | 5.39 | 1.30 (0.52 - 2.67) | 24 | 25.18 | 0.95 (0.61 - 1.42) |
| **Female Reproductive Organs** | 8 | 9.65 | 0.83 (0.36 - 1.63) | <3 | 1.61 | 1.24 (0.15 - 4.48) | <3 | 1.68 | 1.19 (0.14 - 4.29) | 5 | 7.91 | 0.63 (0.21 - 1.47) |
| **Urinary Tract** | <3 | 2.45 | 0.41 (0.01 - 2.27) | 0 | 0.40 | - | 0 | 0.43 | - | <3 | 2.00 | 0.50 (0.01 - 2.78) |
| **Brain And Other CNS** | <3 | 1.24 | 1.61 (0.20 - 5.82) | 0 | 0.21 | - | 0 | 0.21 | - | <3 | 1.02 | 1.96 (0.24 - 7.10) |
| **Thyroid And Other Endocrine Glands** | 3 | 3.35 | 0.89 (0.18 - 2.62) | 0 | 0.60 | - | 0 | 0.57 | - | 3 | 2.78 | 1.08 (0.22 - 3.15) |
| **Unknown Site** | <3 | 1.46 | 0.68 (0.02 - 3.80) | 0 | 0.23 | - | 0 | 0.25 | - | <3 | 1.19 | 0.84 (0.02 - 4.67) |
| **Lymphoid, Haematopoietic & Related**  **Tissue** | 6 | 6.21 | 0.97 (0.35 - 2.10) | 0 | 1.03 | - | 0 | 1.08 | - | 6 | 5.08 | 1.18 (0.43 - 2.57) |
| **Other Cancers** | <3 | 2.39 | 0.84 (0.10 - 3.02) | 0 | 0.39 | - | <3 | 0.41 | - | <3 | 1.96 | 0.51 (0.01 - 2.85) |
| **All Malignancies** | 95 | 88.76 | 1.07 (0.87 - 1.31) | 16 | 14.94 | 1.07 (0.61 - 1.74) | 13 | 15.52 | 0.84 (0.45 - 1.43) | 82 | 72.82 | 1.13 (0.90 - 1.40) |

+ Refer to Table S2 for ICD-10 classification of cancer categories.

**Table 4** **Comparison of cancer incidence rates amongst 6207 eligible male mine/refinery workers, to the Australian general population presented by duration of employment in maintenance/production**

|  | **No EMPLOYMENT IN PRODUCTION OR MAINTENANCE** | | | | **DURATION OF EMPLOYMENT IN PRODUCTION OR MAINTENANCE** | | | | | | | | | | |
| --- | --- | --- | --- | --- | --- | --- | --- | --- | --- | --- | --- | --- | --- | --- | --- |
|  |  |  |  |  | **3 months to < 10 years** | | | | **10- 20 years** | | | | **> 20 years** | | |
| **Cancer categories** | | **O** | **E** | **SIR (95% CI)** | | **O** | **E** | **SIR (95% CI)** | | **O** | **E** | **SIR (95% CI)** | **O** | **E** | **SIR (95% CI)** |
| **Lip, Oral Cavity and Pharynx** | | 5 | 8.91 | 0.56 (0.18 - 1.31) | | 21 | 14.98 | 1.40 (0.87 - 2.14) | | 12 | 15.02 | 0.80 (0.41 - 1.40) | 10 | 14.60 | 0.68 (0.33 - 1.26) |
| Lip | | 4 | 2.92 | 1.37 (0.37 - 3.51) | | 11 | 5.39 | 2.04 (1.02 - 3.65) | | 5 | 5.06 | 0.99 (0.32 - 2.31) | 4 | 4.09 | 0.98 (0.27 - 2.51) |
| Pharynx | | 0 | 2.53 | - | | 5 | 4.14 | 1.21 (0.39 - 2.82) | | 3 | 4.20 | 0.71 (0.15 - 2.09) | <3 | 4.44 | 0.23 (0.01 - 1.25) |
| **Digestive Organs** | | 39 | 42.45 | 0.92 (0.65 - 1.26) | | 52 | 56.31 | 0.92 (0.69 - 1.21) | | 61 | 71.79 | 0.85 (0.65 - 1.09) | 67 | 76.26 | 0.88 (0.68 - 1.12) |
| Oesophagus | | <3 | 2.93 | 0.68 (0.08 - 2.47) | | 6 | 3.88 | 1.55 (0.57 - 3.37) | | 4 | 4.89 | 0.82 (0.22 - 2.10) | 6 | 5.42 | 1.11 (0.41 - 2.41) |
| Stomach | | 4 | 4.50 | 0.89 (0.24 - 2.28) | | <3 | 5.91 | 0.34 (0.04 - 1.22) | | 8 | 7.88 | 1.02 (0.44 - 2.00) | 8 | 7.56 | 1.06 (0.46 - 2.09) |
| Colorectal | | 28 | 26.11 | 1.07 (0.71 - 1.55) | | 31 | 34.35 | 0.90 (0.61 - 1.28) | | 35 | 44.51 | 0.79 (0.55 - 1.09) | 43 | 46.60 | 0.92 (0.67 - 1.24) |
| Colon | | 15 | 15.47 | 0.97 (0.54 - 1.60) | | 13 | 19.70 | 0.66 (0.35 - 1.13) | | 21 | 26.56 | 0.79 (0.49 - 1.21) | 21 | 27.34 | 0.77 (0.48 - 1.17) |
| Rectum | | 13 | 10.15 | 1.28 (0.68 - 2.19) | | 18 | 13.88 | 1.30 (0.77 - 2.05) | | 14 | 17.17 | 0.82 (0.45 - 1.37) | 22 | 18.39 | 1.20 (0.75 - 1.81) |
| Liver | | <3 | 3.08 | 0.65 (0.08 - 2.34) | | 5 | 4.54 | 1.10 (0.36 - 2.57) | | 4 | 4.78 | 0.84 (0.23 - 2.14) | <3 | 6.02 | 0.33 (0.04 - 1.20) |
| Gallbladder | | <3 | 1.00 | 1.00 (0.03 - 5.59) | | <3 | 1.25 | 0.80 (0.02 - 4.45) | | 4 | 1.71 | 2.34 (0.64 - 5.99) | <3 | 1.76 | 0.57 (0.01 - 3.16) |
| Pancreas | | <3 | 4.07 | 0.25 (0.01 - 1.37) | | 6 | 5.24 | 1.15 (0.42 - 2.49) | | 6 | 6.82 | 0.88 (0.32 - 1.91) | 5 | 7.47 | 0.67 (0.22 - 1.56) |
| **Respiratory and Intrathoracic Organs** | | 13 | 23.63 | 0.55 (0.29 - 0.94) | | 29 | 28.69 | 1.01 (0.68 - 1.45) | | 37 | 41.61 | 0.89 (0.63 - 1.23) | 27 | 40.92 | 0.66 (0.43 - 0.96) |
| Larynx | | <3 | 2.13 | 0.94 (0.11 - 3.39) | | <3 | 2.84 | 0.71 (0.09 - 2.55) | | <3 | 3.80 | 0.53 (0.06 - 1.90) | <3 | 3.62 | 0.28 (0.01 - 1.54) |
| Lung | | 11 | 20.93 | 0.53 (0.26 - 0.94) | | 25 | 24.90 | 1.00 (0.65 - 1.48) | | 34 | 36.85 | 0.92 (0.64 - 1.29) | 25 | 36.40 | 0.69 (0.44 - 1.01) |
| **Melanoma** | | 28 | 22.23 | 1.26 (0.84 - 1.82) | | 52 | 38.31 | 1.36 (1.01 - 1.78) | | 46 | 36.40 | 1.26 (0.93 - 1.69) | 37 | 36.15 | 1.02 (0.72 - 1.41) |
| **Mesothelioma** | | <3 | 1.68 | 1.19 (0.14 - 4.30) | | 11 | 1.94 | 5.68 (2.83 - 10.16) | | 4 | 2.89 | 1.38 (0.38 - 3.54) | 6 | 3.03 | 1.98 (0.73 - 4.31) |
| **Male Reproductive Organs** | | 75 | 55.90 | 1.34 (1.06 - 1.68) | | 69 | 75.69 | 0.91 (0.71 - 1.15) | | 73 | 86.60 | 0.84 (0.66 - 1.06) | 133 | 113.85 | 1.17 (0.98 - 1.38) |
| Prostate | | 73 | 53.47 | 1.37 (1.07 - 1.72) | | 65 | 68.63 | 0.95 (0.73 - 1.21) | | 69 | 82.90 | 0.83 (0.65 - 1.05) | 132 | 111.99 | 1.18 (0.99 - 1.40) |
| Testis | | <3 | 2.05 | 0.49 (0.01 - 2.71) | | 3 | 6.56 | 0.46 (0.09 - 1.34) | | 4 | 3.08 | 1.30 (0.35 - 3.32) | <3 | 1.29 | 0.78 (0.02 - 4.33) |
| **Urinary Tract** | | 12 | 12.03 | 1.00 (0.52 - 1.74) | | 19 | 16.09 | 1.18 (0.71 - 1.84) | | 15 | 20.53 | 0.73 (0.41 - 1.21) | 24 | 20.96 | 1.15 (0.73 - 1.70) |
| Kidney | | 4 | 5.70 | 0.70 (0.19 - 1.80) | | 9 | 8.73 | 1.03 (0.47 - 1.96) | | 5 | 9.30 | 0.54 (0.17 - 1.25) | 13 | 10.45 | 1.24 (0.66 - 2.13) |
| Bladder | | 7 | 5.58 | 1.25 (0.50 - 2.58) | | 10 | 6.47 | 1.55 (0.74 - 2.84) | | 9 | 9.92 | 0.91 (0.41 - 1.72) | 10 | 9.21 | 1.09 (0.52 - 2.00) |
| **Brain and Other CNS** | | <3 | 3.18 | 0.31 (0.01 - 1.75) | | 3 | 5.70 | 0.53 (0.11 - 1.54) | | 9 | 5.27 | 1.71 (0.78 - 3.24) | 4 | 4.97 | 0.81 (0.22 - 2.06) |
| Brain | | <3 | 3.06 | 0.33 (0.01 - 1.82) | | 3 | 5.45 | 0.55 (0.11 - 1.61) | | 9 | 5.07 | 1.78 (0.81 - 3.37) | 4 | 4.81 | 0.83 (0.23 - 2.13) |
| **Thyroid and Other Endocrine Glands** | | 9 | 1.68 | 5.35 (2.44 - 10.15) | | 5 | 3.40 | 1.47 (0.48 - 3.44) | | 3 | 2.63 | 1.14 (0.24 - 3.33) | 4 | 2.77 | 1.44 (0.39 - 3.69) |
| Thyroid | | 8 | 1.55 | 5.15 (2.22 - 10.14) | | 5 | 3.13 | 1.60 (0.52 - 3.73) | | 3 | 2.42 | 1.24 (0.26 - 3.62) | 4 | 2.59 | 1.55 (0.42 - 3.96) |
| **Unknown Site** | | 5 | 4.73 | 1.06 (0.34 - 2.46) | | 4 | 5.84 | 0.69 (0.19 - 1.75) | | 6 | 8.57 | 0.70 (0.26 - 1.52) | 4 | 7.50 | 0.53 (0.15 - 1.37) |
| **Lymphoid, Haematopoietic & Related Tissue** | | 16 | 17.17 | 0.93 (0.53 - 1.51) | | 23 | 26.69 | 0.86 (0.55 - 1.29) | | 16 | 28.30 | 0.57 (0.32 - 0.92) | 23 | 29.22 | 0.79 (0.50 - 1.18) |
| Hodgkins | | 0 | 0.82 | - | | 0 | 2.07 | - | | <3 | 1.25 | 0.80 (0.02 - 4.45) | 0 | 0.96 | - |
| Non-Hodgkin Lymphoma | | 5 | 8.03 | 0.62 (0.20 - 1.45) | | 14 | 12.74 | 1.10 (0.60 - 1.84) | | 8 | 13.21 | 0.61 (0.26 - 1.19) | 12 | 13.69 | 0.88 (0.45 - 1.53) |
| Diffuse Non-Hodgkins Lymphoma | | <3 | 4.10 | 0.49 (0.06 - 1.76) | | 7 | 6.33 | 1.11 (0.44 - 2.28) | | 4 | 6.67 | 0.60 (0.16 - 1.54) | 8 | 7.25 | 1.10 (0.48 - 2.17) |
| Multiple Myeloma | | 4 | 2.59 | 1.54 (0.42 - 3.95) | | 3 | 3.45 | 0.87 (0.18 - 2.54) | | <3 | 4.28 | 0.23 (0.01 - 1.30) | 4 | 4.79 | 0.83 (0.23 - 2.14) |
| Leukaemia | | 7 | 5.50 | 1.27 (0.51 - 2.62) | | 5 | 8.12 | 0.62 (0.20 - 1.44) | | 6 | 9.19 | 0.65 (0.24 - 1.42) | 7 | 9.37 | 0.75 (0.30 - 1.54) |
| Acute Lymphoid Leukaemia | | 3 | 2.88 | 1.04 (0.21 - 3.04) | | 3 | 4.15 | 0.72 (0.15 - 2.11) | | 3 | 4.75 | 0.63 (0.13 - 1.85) | 4 | 5.19 | 0.77 (0.21 - 1.97) |
| Myeloid Leukaemia | | <3 | 2.00 | 1.00 (0.12 - 3.62) | | <3 | 3.24 | 0.62 (0.07 - 2.23) | | <3 | 3.37 | 0.59 (0.07 - 2.14) | <3 | 3.10 | 0.65 (0.08 - 2.33) |
| **Other Cancers** | | 4 | 7.24 | 0.55 (0.15 - 1.41) | | 6 | 10.98 | 0.55 (0.20 - 1.19) | | 8 | 11.89 | 0.67 (0.29 - 1.33) | 3 | 11.88 | 0.25 (0.05 - 0.74) |
| Connective Tissue | | 0 | 1.25 | - | | <3 | 2.21 | 0.45 (0.01 - 2.52) | | <3 | 2.05 | 0.49 (0.01 - 2.72) | <3 | 1.93 | 0.52 (0.01 - 2.89) |
| Myelodysplastic | | <3 | 1.59 | 1.26 (0.15 - 4.54) | | 0 | 1.61 | - | | 3 | 2.66 | 1.13 (0.23 - 3.30) | <3 | 2.84 | 0.35 (0.01 - 1.96) |
| **All Malignancies** | | 209 | 200.83 | 1.04 (0.90 - 1.19) | | 294 | 284.61 | 1.03 (0.92 - 1.16) | | 290 | 331.50 | 0.87 (0.78 - 0.98) | 342 | 362.11 | 0.94 (0.85 - 1.05) |

+ Refer to Table S2 for ICD-10 classification of cancer categories.
